# scSemiAAE: a semi-supervised clustering model for single-cell RNA-seq data

**DOI:** 10.1186/s12859-023-05339-4

**Published:** 2023-05-26

**Authors:** Zile Wang, Haiyun Wang, Jianping Zhao, Chunhou Zheng

**Affiliations:** 1 School of Mathematics and System Science, Xinjiang University, Urumqi, China; 2 School of Computer Science and Technology, Anhui University, Hefei, China

**Keywords:** Deep learning, scRNA-seq, Semi-supervised, Clustering, Adversarial autoencoder

## Abstract

**Background:**

Single-cell RNA sequencing (scRNA-seq) strives to capture cellular diversity with higher resolution than bulk RNA sequencing. Clustering analysis is critical to transcriptome research as it allows for further identification and discovery of new cell types. Unsupervised clustering cannot integrate prior knowledge where relevant information is widely available. Purely unsupervised clustering algorithms may not yield biologically interpretable clusters when confronted with the high dimensionality of scRNA-seq data and frequent dropout events, which makes identification of cell types more challenging.

**Results:**

We propose scSemiAAE, a semi-supervised clustering model for scRNA sequence analysis using deep generative neural networks. Specifically, scSemiAAE carefully designs a ZINB adversarial autoencoder-based architecture that inherently integrates adversarial training and semi-supervised modules in the latent space. In a series of experiments on scRNA-seq datasets spanning thousands to tens of thousands of cells, scSemiAAE can significantly improve clustering performance compared to dozens of unsupervised and semi-supervised algorithms, promoting clustering and interpretability of downstream analyses.

**Conclusion:**

scSemiAAE is a Python-based algorithm implemented on the VSCode platform that provides efficient visualization, clustering, and cell type assignment for scRNA-seq data. The tool is available from https://github.com/WHang98/scSemiAAE.

**Supplementary Information:**

The online version contains supplementary material available at 10.1186/s12859-023-05339-4.

## Introduction

With the boom in sequencing techniques, single-cell transcriptome sequencing quantifies gene expression levels at the resolution of individual cells, providing new insights into the internal heterogeneity of cellular tissues [1]. Clustering is a key link in single-cell transcriptional profiling and plays an important role in revealing cell subtypes, dividing gene sequences, and inferring cell lineages. Traditional clustering methods are mainly divided into density-based clustering, neural network, ensemble learning, k-means, mixture model, graph-based clustering and hierarchical clustering [1]. Responding to the dimensional catastrophe [2] and the explosive growth of sample volumes caused by scRNA-seq [3–5], early clustering studies often combined PCA [6], t-SNE [7, 8], UMAP [9] and other dimensionality reduction methods to complete cell grouping and visualization, including pcaReduce [10], TooManyCells [11] and Seurat [12], etc. However, scRNA-seq data is often sparse and noisy [13] due to a complex combination of biological variability and technological reasons. Traditional clustering methods ignore the extreme sparsity of gene expression in single-cell transcriptome sequences, thus cannot achieve ideal clustering results with basic dimensionality reduction methods alone [3].

Relying on single-cell sequencing technology, researchers have access to large-scale sample data, which provides a unique development opportunity for the application of deep learning. scDeepCluster [14, 15] employs an autoencoder with the Zero-Inflated Negative Binomial (ZINB) [16] distribution to simultaneously reduce dimensionality and denoise the data, and then uses a deep embedding clustering algorithm to identify cell types for the data in the bottleneck layer. scGAE [17] provides a new perspective for exploiting the information between cells and genes by building K-Nearest Neighbor (KNN) [18] graphs, considering count matrices and adjacency matrices as the input to the autoencoder. However, the huge computational effort involved in erecting the adjacency matrix makes this approach stretch in applications with large-scale data. scDSC [19] combines Convolutional Neural Network (CNN) [20, 21] and an autoencoder in a self-supervised manner to further explore the fusion of spatial structure of cells and intergenic information. Variational Autoencoders (VAEs) [22] apply the Kullback-Leibler (KL) divergence penalty to impose a prior distribution (usually a Gaussian distribution) on the hidden code vectors of the autoencoder. scDHA [23] is a stacked Bayesian self-learning network based on VAEs that projects data into multiple low-dimensional spaces. Although VAEs perform well in generative models, the regularization based on the KL loss restricts the setting of the prior distribution. Generative Adversarial Networks (GANs) [24] firstly introduce adversarial training to directly shape the output distribution of the network through Generative Moment Matching Network (GMMN) [25], which allows for alternative choices for the prior distribution. scDEC [26] models the data based on a symmetric GAN structure, jointly optimizing latent feature representation learning and cell clustering. Recently, Adversarial Autoencoders (AAEs) [27] have been proposed, which combine the advantages of probabilistic autoencoders and generative adversarial networks to perform variational inference by matching the aggregated posteriors of the hidden code vectors of the autoencoder to an arbitrary prior distribution. ​It has been shown that this approach achieves better results in reconstructing samples and image classification [27, 28].

​Many downstream analyses, such as differential expression, trajectory inference, etc., rely on the initial clustering results, which require the clustering results to be biologically interpretable. Due to the lack of support from prior information, unsupervised clustering sometimes fails to yield meaningful clusters consistent with prior knowledge. Consequently, the user often needs to repeatedly adjust the cluster parameters manually until a satisfactory cluster is found. We note that prior knowledge is widely available in many cases. A considerable number of cell type information have been published, such as Montoro [29], Puram [30]. Taking advantage of the prior information can avoid suboptimal or illogical clustering results to some extent.

Recently, some semi-supervised clustering algorithms have been proposed, such as scDCC [31], scSemiAE [32], and ItClust [33] etc. scDCC converts partial prior knowledge into pairwise constraints and adds them as additional terms in the loss to guide the deep learning model to better learn latent representations. However, this algorithm constructs soft pairwise constraints with some subjectivity and needs more prior information to define each cell one by one. scSemiAE predicts cell types through a classifier and transfers partial labels information to an autoencoder for fine tuning. It is not difficult to find that the performance of the classifier has a great influence on learning features, and the latent space has no regularization constraints, which may cause overfitting. ItClust performs supervised learning and cell classification on scRAN-seq data, exploiting cell type-specific gene expression information from the source data. However, the quality and quantity of the reference data can highly affect the training and clustering results of the target dataset.

Here, we propose a more flexible framework of semi-supervised clustering, scSemiAAE, which carefully designs a ZINB loss-based autoencoder architecture that inherently integrates adversarial training and semi-supervised modules in the latent space. The study indicates that we can guide the model to obtain a better latent representation by a small portion of label information, and then get more accurate clustering results. A series of experiments on spanning multiple datasets show that scSemiAAE outperforms published unsupervised and semi-supervised clustering tools of clustering accuracy and scalability.

## Methods

### Workflow of the scSemiAAE model

#### Dataset information

The proposed scSemiAAE method is evaluated on nine real scRNA-seq datasets. Table 1 provides an overview of the specific properties of these datasets. The 10X PBMC [34], Human kidney cells [35], Human liver [36], Tabula Muris [37] and Karagiannis [38] datasets are provided by the 10X Genomics scRNA-seq platform. For the Worm neuron cells [39] dataset, the author analyzes approximately 50,000 cells from L2 larval stage of Caenorhabditis elegans and identified cell types. The CITE-seq PBMC [40] dataset is divided into 15 clusters by cluster analysis and gene differential expression analysis. The Shekhar mouse retina cells [41] and Baron(human) [42] datasets are provided by the Drop-seq and inDrop platforms, respectively.

**Table 1:** The information of datasets

Dataset	Cells	Genes	Class	Organ/tissue	Platform
10X PBMC	4271	16,449	8	Human peripheral blood mononuclear cells	10X Genomics
Worm neuron cells	4186	11,955	10	Worm neuron cells	sci-RNA-seq
Human kidney cells	5685	25,215	11	Human kidney cells	10X Genomics
CITE-seq PBMC	8671	18,677	15	Human peripheral blood mononuclear cells	ECCITE-seq
Human liver	8444	20,007	11	Human liver	10X Genomics
Baron(human)	8569	20,215	14	Human pancreas	inDrop
Shekhar mouse retina	27,499	13,166	19	Mouse retina	Drop-seq
Tabula Muris	54,439	23,432	40	Mouse organs	10X Genomics
Karagiannis	72,914	19,011	12	Human blood	10X Genomics

**Fig. 1:**
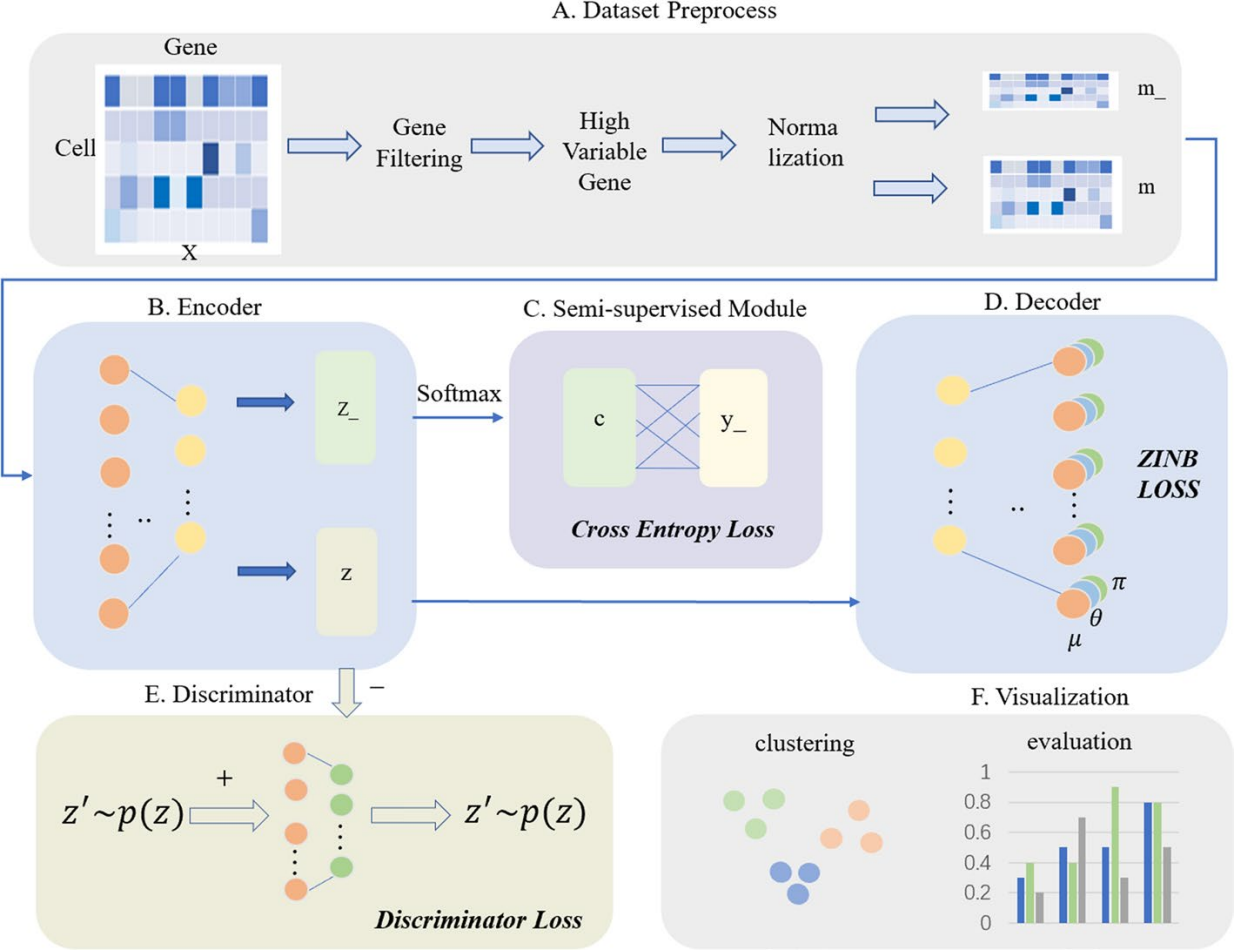
The illustration of scSemiAAE model. **A** The scRNA-seq count matrix *X* is preprocessed through gene filtering, screening of highly variable genes, and normalization. Next, it is divided into *m_* and *m* depending on whether it contains true labels. **B** The encoder receives *m_* and *m* to generate the corresponding latent variables *z_* and* z*, respectively. **C** The SoftMax layer transforms the latent vector *z_* into the pseudo-label *c*, which is then combined with the partial true label *y_* to create a cross-entropy loss. **D** The decoder reconstructs the potential representation *z* with a zero-inflated negative binomial loss constraint. **E** Simultaneously, the latent feature *z* is fed to the discriminator for adversarial training, comprising the discriminator loss. **F** After completing training process, all the latent *z* and labels *c* are concatenated, and the final clustering results are given by a Gaussian mixture model

The scSemiAAE for scRNA-seq data analysis is mainly consists of three computational modules (Fig. 1). The first section is an autoencoder based on the ZINB model, which provides a low-dimensional representation. The second module constructs cross-entropy [34] that introduces label information into the latent space for semi-supervised learning. The third part builds a discriminator network to distinguish between “real” and “fake”samples. Moreover, we use the encoder of the autoencoder as a generator network for making "fake" samples. The details of each step are described as follows.

### Data preprocessing

Raw single cell RNA sequence count data $X$, where rows indicate cells and columns point to genes, is preprocessed by the Python package SCANPY [43]. Firstly, genes that are not counted in the cell are filtered. Secondly, the size factor is calculated, and the read counts are normalized. We denote the total number of RNA molecules per cell as $n_{i}$, and its corresponding median as ${\text{med}}\left( {n_{i} } \right)$, thus the size factor of the cell $i$ is ${\text{sf}}_{i} = n_{i} /{\text{med}}(n_{i} )$. Let the ${\text{j}}_{th}$ gene expression value of the $i_{th}$ cell of the input matrix $X$ be $x_{ij}$ and the normalized expression be $x^{^{\prime}}_{ij} = x_{ij} /{\text{sf}}_{i}$. Finally, to prevent domination by highly expressed genes and features, we log-transform the value $l_{ij} = \log_{2} (x^{^{\prime}}_{ij} + 1)$, and scale the counts $m_{ij} = (l_{ij} - {\text{mean}}_{j} )/{\text{std}}_{j}$. Here, ${\text{mean}}_{j}$ and ${\text{std}}_{j}$ are the mean and standard deviation of the logarithmic expression values of ${\text{j}}_{th}$ gene in all cells, respectively. The processed matrix $M$, consisting of elements $m_{ij}$, is fed to subsequent cluster analysis.

### Autoencoder based on ZINB model

In this paper, we apply a denoising autoencoder based on ZINB loss to capture the features of single cell RNA sequences. We first corrupt the processed matrix with random Gaussian noise, then map the read counted input to the embedding space for clustering.

$$\hat{M} = C(M),$$

1 $$Z = E_{\varphi } (\hat{M}),$$

$$P = D_{\phi } (E_{\varphi } (\hat{M})).$$

Typically, the matrix $M$ is corrupted by $C(M) = M + \lambda \delta$, $\delta$ denotes random Gaussian noise, $\lambda$ corresponds to its coefficient. Each layer of the encoder $E$ function can be expressed as $f_{E} (m) = {\text{mW}}_{E} + b_{E}$, and each layer of the decoder $D$ function can be represented as $f_{D} \left( z \right) = zW_{D} + b_{D}$, where $W$ is the weight matrix and $b$ is the bias vector. The encoder represents the data in a low-dimensional space and thus gets the latent layer $z$, while the decoder tries to reconstruct from the compressed data. Theoretically, optimizing this procedure can lead to a condensed version of the primitive high-dimensional form. Unlike conventional autoencoders, the decoder does not perform reconstruction and only gives the ZINB distribution parameters $P$. In this regard, we attach three separate fully connected layer on the last hidden layer $f_{D^{\prime}} \left( {z^{\prime}} \right)$ of the decoder.

$${\text{Mean}} = \exp (W_{u} D) \times {\text{diag}}({\text{sf}}),$$

2 $${\text{Disp = }}\exp (W_{u} D),$$

$${\text{Dropout}} = sig\bmod (W_{\theta } D).$$

where $sf$ refers to the size factor, $D$ indicates the decoder function, $sigmod$ means the activation function, $W_{M}$,$W_{\pi }$ and $W_{\theta }$ represent the parameters weights to be learned in the last three fully connected layers, respectively.

3 $${\text{NB}}(X|u,\theta ) = \frac{\Gamma (X + \theta )}{{X!\Gamma (\theta )}}\left( {\frac{\theta }{\theta + u}} \right)^{\theta } \left( {\frac{u}{\theta + u}} \right)^{X} ,$$

4 $$\begin{gathered} {\text{ZINB}}(X|u,\pi ,\theta ) = \pi \delta_{0} \left( x \right) + \left( {1 - \pi } \right){\text{NB}}(x|u,\theta ). \hfill \\ \hfill \\ \end{gathered}$$

A negative binomial distribution, with mean $u$, the dispersion $\theta$, and the additional coefficient of the zero-probability point quality weight $\pi$ (probability of dropout events), parameterizes the ZINB loss. Notably, the distribution is calculated using the original gene count matrix $X$.

5 $$\begin{gathered} L_{{{\text{zinb}}}} = \sum { - \log ({\text{ZINB}}(X|u,\pi ,\theta ))} \hfill \\ \hfill \\ \end{gathered}$$

### Semi-supervised module

Cross-entropy [44] originates from Shannon's information theory. It is often represented as the difference between the predicted probability distribution and the true probability distribution in model learning. The smaller the value of cross-entropy, the more accurate the prediction of the model. Considering the proportion of labeled samples, the clustering approaches can be chosen flexibly. When only a small portion of the label information is available, the labeled samples $m_{ - }$ are first separated, corrupted, and then fed into the encoder to produce the corresponding latent variables $z_{ - }$. Subsequently, the partial latent features are passed through the SoftMax layer to generate the pseudo-labels $c$, which construct the cross-entropy loss with the true labels $y_{\_}$. Here, we adopt 20%-25% of the true label information to guide the model learning parameters.

6 $$L_{{{\text{ce}}}} = - \frac{1}{S}\sum\nolimits_{i = 0}^{S - 1} {y_{\_i} \log c_{i} + (1 - y_{\_i} )} \log (1 - c_{i} ).$$

The number of label information used for semi-supervision $S$, the true cell labels $y_{\_}$, and the pseudo-labels $c$, constitute the cross-entropy loss. Note that the true cell labels $y_{\_}$ are not cell types, but simply represent information about which class a cell belongs to.

### Discriminator and Generator Networks

Generative adversarial network (GAN) usually iteratively training the generative model $g\varphi \left( {t|s} \right)$ and the discriminative model $d\chi \left( t \right)$ to realize the adversarial training [9]. We feed samples from the generator (“fake” samples) and the target distribution (“true” samples) into a discriminative model for training to correctly predict whether a sample is “true” or “fake”. The generative model takes $s$ as input, extracted from the selected prior distribution $p\left( s \right)$. To fool the discriminative model [24], it continues training until the generated samples $t$ are indistinguishable from the target samples $t$. The following minimax function [9] can accomplish the target:

7 $$\mathop {\min }\limits_{g} \mathop {\max }\limits_{d} E_{t\sim f(t)} [\log d_{\chi } (t)] + E_{{t\sim g_{\phi } (t|s)}} [\log (1 - d_{\chi } (t))].$$

It turns out that, for ideal discriminative models, optimizing the generator equal to minimizing the Jensen-Shannon divergence between the generative distribution and the target distribution [9]. Overall, it is rational to presume the discriminator rapidly reaches optimal performance during training [9]. Further, we could bypass the complicated Jensen-Shannon divergence calculation and thus learn the distribution easily.

To prevent the model from overfitting, we impose regularization constraints on the latent space by adversarial training. A discriminative network is trained to divide potential samples from $p\left( z \right)$ and $q_{\varphi } \left( {z|m} \right)$.The latter is both a probabilistic encoder in the autoencoder and a generative model in the adversarial framework. The loss for training the discriminator $d_{\chi } \left( z \right)$ is:

8 $$L_{{{\text{dis}}}} = - \frac{1}{n}\sum\nolimits_{i = 0}^{n - 1} {\log d_{\chi } (z_{{{\text{true}}_{i} }} )} - \frac{1}{n}\sum\nolimits_{j = n}^{2n - 1} {\log d_{{_{\chi } }} (1 - z_{{{\text{fake}}_{j} }} )} .$$

where $z_{{true_{i} }} = 0:n - 1 \sim p\left( z \right)$, $z_{{fake_{j} }} = n:2n - 1 \sim q_{\varphi } \left( {z|m} \right)$, $d$ represents the discriminator, and *n* is the size of the training batch, $p(z) = N(u,\Sigma )$.

We consider the encoder of the autoencoder as the generator and the latent vectors $z$ as the generated samples. A set of vectors with the same dimension are drawn from a multivariate Gaussian distribution as the true samples, and the generator loss is constructed as follows:

9 $$L_{{{\text{ge}}}} = - \frac{1}{n}\sum\nolimits_{i = 0}^{n - 1} {\log d_{\lambda } (z_{{{\text{fake}}_{i} }} )} .$$

where $z_{{fake_{i} }} = 0:n - 1 \sim q_{\varphi } \left( {z|m} \right)$, $n$ refers to the training batch size. As the two losses continue to optimize, the distribution of generated samples is constantly moving to the target distribution of the generative model. In this case, the discriminator is maximally puzzled and cannot differentiate between "true" and "fake" samples.

We summarize the training procedure of the algorithm in Table 2 to ensure the completeness and readability of the algorithm.

**Table 2:**
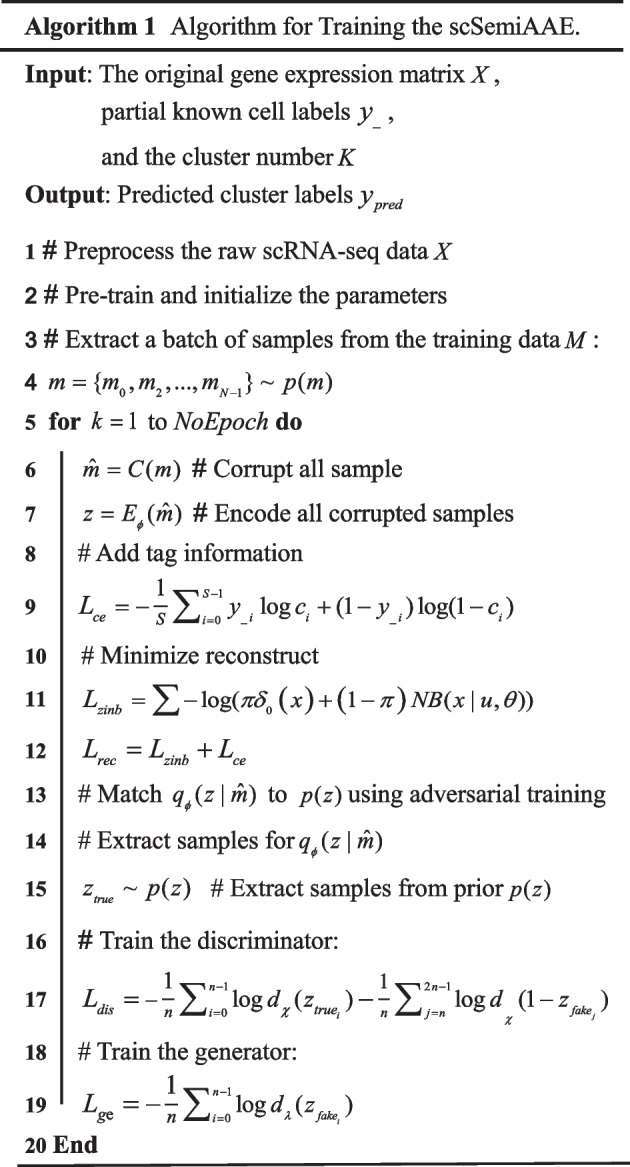
Summary of scSemiAAE algorithm

### Metric of performance evaluation

The paper compares different clustering methods based on multiple metrics such as Adjusted Rand Index (ARI) [45], Normalized Mutual Information (NMI) [46] and Accuracy (ACC) [14].

The Rand index [47] measures the agreement between two cluster assignments, while the ARI corrects for the lack of a constant value when cluster assignments are randomly chosen. The ARI values are in the range [−1, 1]. A value of one indicates perfect grouping. A value of zero shows a random assignment of samples to groups, and negative values point to wrong cluster assignments. We define the following four quantities (1) *p* : the count of target pairs in the same sets in $P$ but varied groups in $Q$ (2) *q* : The count of target pairs in the same sets in $Q$ but varied groups in $P$(3) *m* : the count of target pairs in the same sets in both $P$ and $Q$ (4) *n* : the count of target pairs in varied groups in both $P$ and $Q$.

10 $${\text{ARI}} = \frac{{C_{n}^{2} (m + q) - [(m + n)(m + p) + (p + q)(n + q)]}}{{C_{n}^{2} - [(m + n)(m + p) + (p + q)(n + q)]}}$$

Assume $P$ and $Q$ are true, and predict label assignments given $N$ data points with $U_{P}$ and $U_{Q}$ clusters, respectively. Given two cluster assignments $P$ and $Q$, with $U_{P}$ and $U_{Q}$ clusters on $N$ data points respectively, the NMI is defined as the mutual information between $P$ and $Q$, divided by the entropy of the $P$ and $Q$ clusters. Here, $C_{n}^{2} \,$ is the number of combinations of two elements taken from $n$ elements. The combination is unordered, and it is calculated by $C_{n}^{2} = n\left( {n - 1} \right)/2$.

11 $${\text{NMI}} = \frac{{\sum\nolimits_{m = 1}^{{U_{P} }} {\sum\nolimits_{n = 1}^{{U_{Q} }} {|P_{m} \cap Q_{n} |} \log \frac{{N|P_{m} \cap Q_{n} |}}{{|P_{m} | \times |Q_{n} |}}} }}{{\max \left( { - \sum\nolimits_{m = 1}^{{C_{P} }} {|P_{m} |\log \frac{{|P_{m} |}}{N},\sum\nolimits_{n = 1}^{{C_{Q} }} {|Q_{n} |\log \frac{{|Q_{n} |}}{N}} } } \right)}}.$$

ACC denotes the best match between the predicted cluster and the true cluster. Let $\hat{k}_{i}$and $k_{i}$ be the prediction of the clustering methods and the true label of the data point, ACC is expressed as follows:

12 $${\text{ACC}} = \mathop {\max }\limits_{c} \sum\limits_{i = 1}^{m} 1 \frac{{\{ k_{i} = c(\hat{k}_{i} )\} }}{n}$$

### Implementation

scSemiAAE is implemented in Python 3 (version 3.8.13) using PyTorch [48, 49] (version 1.11.0). In the ZINB model-based autoencoder, the size of the hidden layer is set to (256, 128, 64, 64, 128), where the size of the bottleneck layer is 64. Each layer of the autoencoder adds a dropout of 0.2 and a standard deviation of Gaussian random noise is 1.0. The number of neurons of the discriminator is set to (64, 128, 256, 1).

In the data pre-training stage, the learning rate is set to 0.001, the number of training is 100, the batch size is 128, and the optimizer is Adam [50]. After getting the initialized weights, in the training phase, the algorithm regards the encoder of the autoencoder as the generator, the optimizer selects Adadelta [51], and the parameters are set to rho = 0.95 , lr = 0.01. The discriminator offers Adam as the optimizer, and its parameters are set as the initial learning rate β1 = 0.9, β2 = 0.999, lr = 0.0001. The batch size is set to 128 and the number of training epochs is 100. To coordinate the learning capabilities of the generator and discriminator, we set the discriminator to learn every 5 epochs of training. Cell label usage is set to 20% or 25% for different datasets (Fig. 3C). In addition, for Gaussian mixture clustering to get clustering labels, we give validation results based on experiments (Additional file 1: Figure S2). All experiments are performed on RTX 3060 (16G). The acquisition and implementation of baseline methods and other parameters sensitivity analyses are provided in the Additional file 1.

## Results

### Visualization and accuracy evaluation of different methods

To evaluate the performance of scSemiAAE to distinguish between different cell subpopulations and identify cell types, the research tests on the real scRNA-seq datasets with diverse cell types and numbers. The clustering performances of these methods are evaluated on the basis of (1) whether cell subpopulations could be clearly distinct in the latent space, and (2) whether the clustering results can accurately infer the true cell types. To address the first issue, we apply t-Distributed Stochastic Neighbor Embedding (t-SNE) to project the bottleneck layer into a 2D space to visualize the latent features learned by different methods for scRNA-seq data. To assess the clustering results of different strategies for the second problem, this paper adopts three common metrics, Normalized Mutual Information (NMI), Adjusted Rand Index (ARI), and Accuracy (ACC), based on true cell labels.

**Fig. 2:**
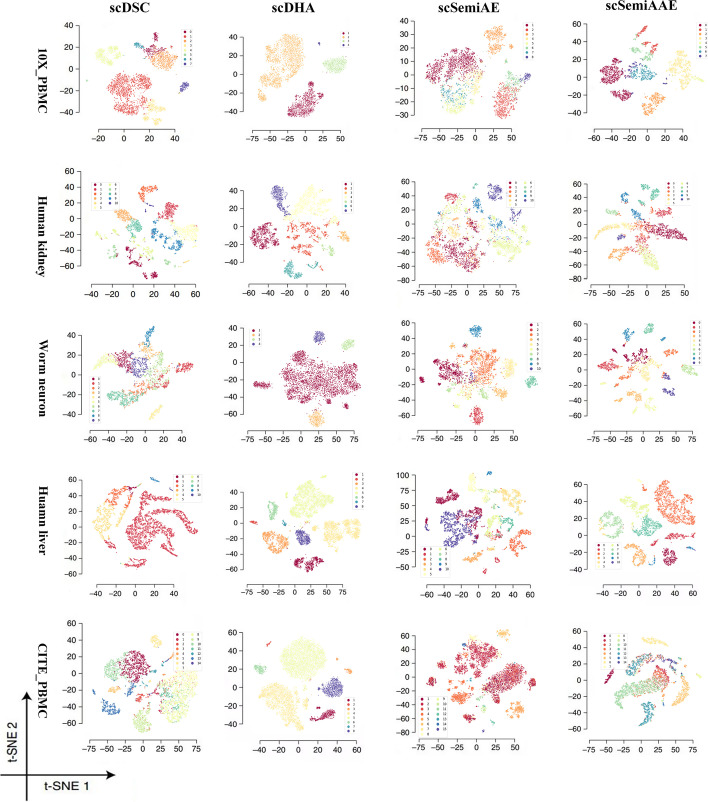
Latent representation visualization. The images base on embedded representations of the 10X_PBMC, Human kidney cells, Worm neuron cells, Human liver and CITE_PBMC datasets. Each dot indicates a cell, and the different colors of the dots point to the predicted labels

As shown in Fig. 2, we select five real datasets of varying complexity for visualization. It is not hard to find that scSemiAAE can achieve ideal separation and clear boundaries for datasets with 8, 10 and 15 different cell subtypes. In differentiation, other methods tend to mix distinct cell subtypes. On the Human kidney dataset, scDHA can also distinguish different types of clusters sparsely compared to scSemiAAE. However, the identified clusters are scattered and incomplete. Cells of various types are mixed in scSemiAE, and it is hard to get satisfactory boundaries between clusters. For example, orange cells and red cells are connected, and the clusters are not dispersed enough. For scDSC, red indicates that cells are distributed over the entire plot on Human liver dataset. We have the identical observation on the 10X_PBMC, Worm neuron cells, and CITE_PBMC datasets. Compared with other clustering methods, scSemiAAE can identify all different cell types, clarify the boundaries between clusters, and ensure the dispersion between clusters.

**Fig. 3:**
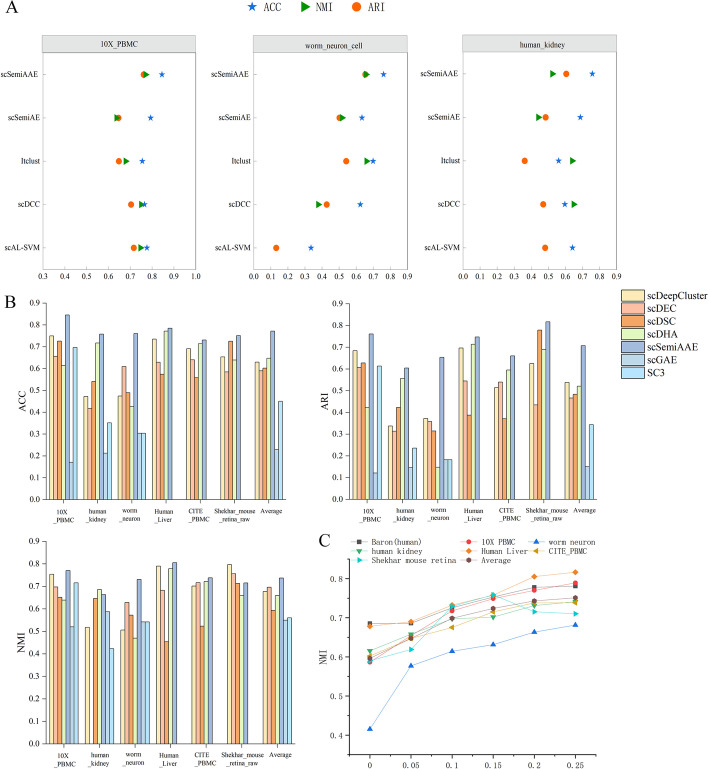
Benchmarking results on real scRNA-seq datasets. Clustering performances of scDeepCluster, scDSC, scDEC, scDHA, SC3, scGAE, scDCC, scAL, Itclust, scSemiAE and scSemiAAE, measured by ACC, NMI and ARI. The first six ones are unsupervised methods, and the remaining ones are semi-supervised clustering algorithms. **A** Comparison with semi-supervised clustering approaches on three datasets with the top 2000 highly scattered genes. **B** The results of unsupervised clustering algorithms. **C** scSemiAAE uses different proportions of labels on seven real datasets, measured by NMI

In terms of consistency of the clustering results with the true labels, we compare scSemiAAE with ten baseline methods, including scDeepCluster [14], scDEC [24], scDSC [17], scDHA [21], SC3 [52], scGAE [16], scDCC [29], Itclust [33],scAL [46], scSemiAE [30]. Notice that the first six ones are unsupervised methods, and the remaining ones are semi-supervised clustering algorithms. Figure 3A and B show the two partitioning techniques, respectively. Apparently, our model significantly surpasses current deep clustering methods on the 10X_PBMC, Human kidney cells, Worm neuron cells, Shekhar mouse retina raw cells, and CITE_PBMC datasets, and slightly better on the Human Liver dataset. For the Worm neuron dataset, scSemiAAE significantly raises ACC by 6.12%, NMI by 7.08%, and ARI by 11.20% compared to the suboptimal metrics of all algorithms. On the 10X_PBMC dataset, scSemiAAE greatly improves by 7.99% on ACC, 2.03% on NMI, and 5.76% on ARI in contrast to the suboptimal metrics of all approaches. The details of the datasets, complete cluster images and indicator comparison are shown in the Additional file 1

### Robustness of scSemiAAE on highly dispersed genes

Most single cell analysis pipelines apply gene filtering strategies to select low variance genes and only keep high dispersion genes (eg. SCANPY). Selecting genes that are highly scattered can enlarge differences between cells but lose critical information between cell populations. To assess the robustness of scSemiAAE to highly scattered genes, we conduct experiments on the top 2000 highly scattered genes in three datasets (Fig. 3A), and then reveal the performance of scSemiAAE and the baseline methods. As the diagram displays, scSemiAAE consistently exceeds other semi-supervised clustering models using full datasets.

### Scalability of scSemiAAE for large-scale datasets

**Fig. 4:**
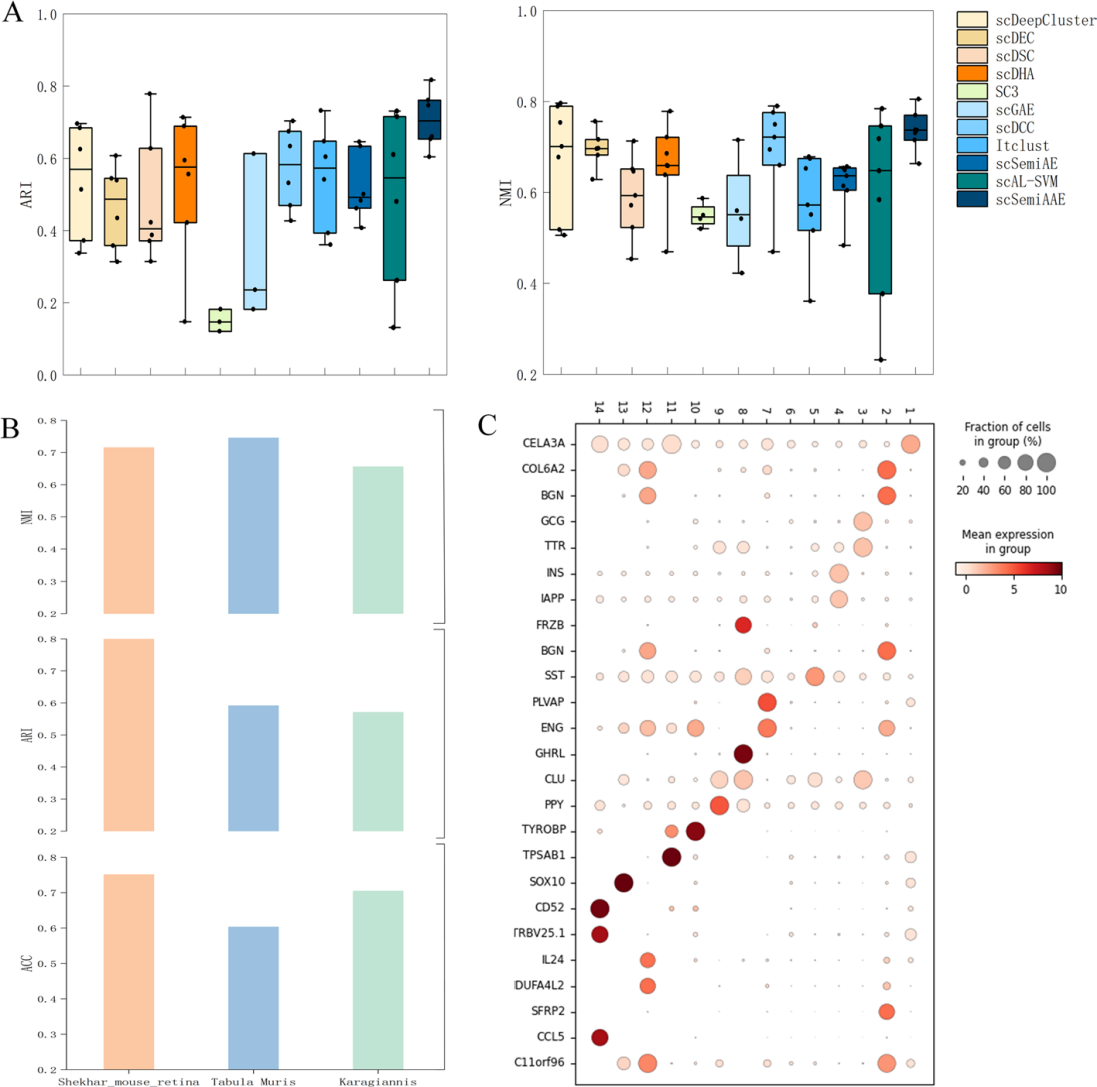
Model performance analysis of scSemiAAE. **A** Comparing the scalability of different algorithms on the real datasets by ARI and NMI metrics. **B** Clustering effects based on large-scale datasets. **C** Differential expression analysis bases on Baron (human) data

The large-scale sample size is one of the main characteristics of single-cell sequencing technology applications, and whether it can handle large-scale data is an important consideration for current clustering algorithms. The experiments on three larger datasets - Shekhar mouse retina raw data, Tabula Muris, and Karagiannis - demonstrate that scSemiAAE is effective for clustering large-scale single-cell transcriptome data. For example, on the Shekhar dataset with 27,466 samples, our algorithm achieved an Adjusted Rand Index (ARI) value of 0.8 or higher compared to the "reference" labels, indicating high consistency (Fig. 3B). Similarly, on the Tabula Muris dataset with 54,439 cells and 40 cell types across 20 organs and tissues, the clustering NMI metric by the method is 0.7456; on the Karagiannis dataset with 72,914 cells, the ACC and NMI metrics also reached above 0.7 (Fig.4B). Overall, these results demonstrate that scSemiAAE performs well on large datasets. In comparison, neither scGAE nor SC3 can handle datasets with more than 8000 samples under the same memory conditions.

Furthermore, we plot boxplots of ARI and NMI metrics for 11 different clustering algorithms on six real datasets to compare the scalability of models. scSemiAAE demonstrates desirable agreement with reference cell labels on different scRNA datasets with sample sizes ranging from thousands to tens of thousands. The highest ARI value exceeds 0.8, and the lowest ARI value is also above 0.6 (Fig. 4A left panel). Relatively, the box lengths of other baseline methods are obviously longer, and their average clustering accuracy is much lower than our algorithm. The NMI boxplot (Fig. 4A right panel) also displays the same characteristics, with the shortest box length and the highest average clustering accuracy.

### Maker genes identification

Gene expression matrix and cluster labels can be used to identify the differentially expressed genes (DEGs) in each cluster. Here, we choose the Baron (human) dataset to extract its gene markers and analyze the relationship between cell groups and cells. For the dataset, the author collects the transcriptomes of beyond 12,000 single pancreatic cells from two mouse strains and four human donors. Cell clusters could correspond to previously identified cell types, including four types of immune cells, exocrine cell types, activated and quiescent stellate cells , rare epsilon cells, Schwann cells and vascular cells [42]. Figure 4C shows the first 2 marker genes of each cluster. As can be seen from the chart, most of the differentially expressed genes selected according to the scSemiAAE cluster labels are involved in significant expression differences between clusters.

## Discussion

Single-cell transcriptome clustering can identify disease-relevant cell types and subpopulations from heterogeneous samples, contributing to further unravel the physiological mechanisms of cells. Among current clustering tools, unsupervised methods are still dominant. However, when the final number of cell classes is not known, it is possible that unsupervised algorithms fail to produce biologically consistent cell clusters. This requires the user to manually iterate the clustering parameters to achieve satisfactory performance. Not surprisingly, for some datasets, we do not always find the right parameters to adjust the results [3, 53].

Therefore, it is particularly important to incorporate prior knowledge into clustering models. Notably, the priori information here can be partial cell types, cell labels, number of classes, marker genes, protein restrictions, etc. In addition, multi-omics sequencing data can equally serve as the prerequisite, such as CITE-seq [54] (simultaneous analysis of single-cell transcriptome and surface proteins) and single-cell ATAC-seq [55]. Researchers choose to introduce different background knowledge depending on the experimental purpose and algorithm design. In this paper, the proposed scSemiAAE employs partially real cell labels as the priori. We give the details of the data and the source of the label information and place these in the Additional file 1.

Furthermore, this paper presents several directions for improving scSemiAAE. First, we can try different adversarial losses when training the discriminator. The conditional adversarial loss (CGAN) [56] concatenates label information and latent variables, and then send them to the discriminator for training. Considering that this loss can make full use of the remaining pseudo-labels, we believe that Gaussian mixture clustering can be removed, and the pseudo-labels can be used as the final clustering results. This simplifies the model on the one hand and integrates latent features generation and clustering on the other hand. Second, if genes and regulatory elements (REs) were added to the scSemiAAE, it might help to further improve the clustering performance. Third, some studies have developed packages for batch effect correction due to the limitations of sequencing technologies, such as SCALEX [57] and Harmony [58]. It makes sense to explore how this data integration analysis can be incorporated into the scSemiAAE model.

​With scSemiAAE, researchers can perform scRNA-seq analysis on cell types or tissues of interest, further revealing the biological meaning behind the features. We hope that scSemiAAE will help discover new cell types and contribute to the understanding of different cell populations.

## Conclusion

In this study, we propose scSemiAAE that adopts a deep generative model to accurately characterize cellular subpopulations for scRNA-seq data. scSemiAAE inherently integrates adversarial training and semi-supervised clustering by carefully designing a ZINB adversarial autoencoder-based architecture. It is a strong and effective tool for scRNA-seq data, including potential layers visualization, cell clustering, differential expression analysis.

A series of experiments show that scSemiAAE could acquire better performance compared to current clustering techniques, since it can capture ideal latent characteristics to promote cell type identification. The studies also prove that scSemiAAE can handle large-scale datasets and shows robustness and noise resistance on genes with high dispersion. In addition, scSemiAAE can well identify differentially expressed genes, which helps to further explain the biological significance of cell type assignment.

## Supplementary Information

**Additional file 1:** The details of the real datasets, implementation of baseline methods and additional tables and plots.

## Data Availability

The datasets in the paper are available at https://github.com/WHang98/scSemiAAE.

## Abbreviations

scRNA-seq: Single-cell RNA sequencing
ZINB: Zero-inflated negative binomial
PCA: Principal component analysis
t-SNE: T-distributed Stochastic Neighbor Embedding
UMAP: Uniform manifold approximation and projection
KNN: K-nearest neighbor
CNN: Convolutional neural network
KL: Kullback-Leibler
VAEs: Variational autoencoders
GANs: Generative adversarial networks
GMMN: Generative moment matching network
AAEs: Adversarial autoencoders
GMM: Gaussian mixture
ARI: Adjusted rand index
NMI: Normalized mutual information
ACC: Accuracy
DEGs: Differentially expressed genes
CGAN: Conditional adversarial network
REs: Regulatory elements
