# Supplementary material for: scSemiAAE: a semi-supervised clustering model for single-cell RNA-seq data
Source: BMC Bioinformatics. 2023 May 26;24:217. doi: 10.1186/s12859-023-05339-4 (PMC10214737; doi:10.1186/s12859-023-05339-4)
Supplement: Supplementary file 1 — Additional file 1: The details of the real datasets, implementation of baseline methods and additional tables and plots. [file 12859_2023_5339_MOESM1_ESM.docx]

**scSemiAAE: A semi-supervised clustering model for single-cell RNA-seq data**

Zile Wang^（1）^, Haiyun Wang^（1）^, Jianping Zhao^(1,*)^  and Chunhou Zheng^（1,2，*）^

^1^School of Mathematics and System Science, Xinjiang University, Urumqi, Xinjiang 830017, China.

^2^School of Computer Science and Technology, Anhui University, Hefei, Anhui 230601, China

**Supplementary**

In this study, we conduct a wide range of experiments, including visual analysis of clustering, scalability studies on large-scale data, robustness on highly dispersed genes, identification of differentially expressed genes, and parameter analysis of model setups. The acquisition of the datasets and implementation of baseline methods, and the comparison of full clustering results are shown as follows.

**Real dataset availability**

The 10X PBMC dataset (4K PBMCs from healthy donors) is provided by a 10X scRNA-seq platform that analyzed the transcriptome of peripheral blood mononuclear cells (PBMCs) from healthy donors. PBMC 4k data were downloaded from the 10X genomics website (<https://support.10xgenomics.com/single-cell-gene-expression/datassets/2.1.0/pbmc4k>). We download filtered gene/cell matrices and cell labels identified by graph-based clustering (method described at [https://support.10xgenomics.com/single-cell-gene-expression/software/pipelines/latest/output/ analysis](https://support.10xgenomics.com/single-cell-gene-expression/software/pipelines/latest/output/%20analysis)).

Human kidney dataset is downloaded from <https://github.com/xuebaliang/scziDesk/tree/master/dataset/Young>. The authors analyze human kidney tumors and normal tissue from fetal, child, and adult kidneys. The dataset contains 5685 cells divided into 11 clusters with 25215 genes per cell.

Shekhar mouse retina cells [(https://scrnaseq-public-datasets.s3.amazonaws.com/scater-objects/shekhar.rds]((https:/scrnaseq-public-datasets.s3.amazonaws.com/scater-objects/shekhar.rds)).The dataset contains 27499 cells divided into 19 clusters with 13166 genes per cell.

Worm neuron cell dataset is analyzed using science-RNA-seq (single-cell combinatorial indexed RNA sequencing). The authors analyze approximately 50,000 cells from L2 larval stage Caenorhabditis elegans and identified cell types (<http://atlas.gs.washington.edu/worm-rna/docs/>).

CITE-seq dataset (<https://github.com/canzarlab/Specter/tree/>). This dataset contains 3762 cells, 18677 genes and 49 protein markers. The dataset is divided into 12 clusters by cluster analysis and gene differential expression analysis.

Human liver dataset (<http://shiny.baderlab.org/#human-liver-atlas>) (<https://github.com/BaderLab/scClustViz>). This dataset contains 8444 cells and 200007 genes. The authors provide clustering results and marker gene tables. The real labels are obtained by using the "labelCellTypes" function from the scClustViz package. As a result, the dataset contains 11 cell types.

Baron Human dataset (<https://www.ncbi.nlm.nih.gov/geo/query/acc.cgi?acc=GSE84133>), provided by the in-Drop platform, the authors analyze normal human pancreatic tissue. The dataset contains 8569 cells, 20125 genes, and 14 cell types.

The Tabula Muris and Karagiannis datasets (http://scdha.tinnguyen-lab.com/) are analyzed through the 10X genomics platform. Muris et al. create a single cell transcriptome data compilation from the model organism, the small house mouse (Mus musculus), containing 20 organs and tissues with more than 100,000 cells. We collect 54,439 cells, 23,432 genes, and 40 cell types. Karagiannis et al. perform single-cell RNA sequencing of peripheral blood single nuclei from opioid-dependent and control individuals. The dataset contains 72,914 cells, 19,011 genes, and 12 cell types.

**Baseline methods**

We compare scSemiAAE to multiple baseline methods including scDeepCluster(<https://github.com/ttgump/scDeepCluster),scDEC>(<https://github.com/kimmo1019/scDEC),scDSC>(<https://github.com/DHUDBlab/scDSC>),scDHA<(https://github.com/duct317/scDHA>),SC3(<https://bioconductor.org/packages/release/bioc/html/SC3.html>),scGAE(<https://github.com/ZixiangLuo1161/scGAE>),scDCC(<https://github.com/ttgump/scDCC>),scAL([https://github.com/ scAL/xianglin226/scAL](https://github.com/%20scAL/xianglin226/scAL)),scSemiAE(<https://github.com/xuebaliang/scSemiCluster>),Itclust(<https://github.com/jianhuupenn/ItClust>).

These packages and APIs developed by the original authors are used for experimentation. In addition to a unified computational environment, we perform the same preprocessing on the raw count matrix of each competing method, following the steps described in the previous work. Also, to enhance the persuasiveness of the comparison with unsupervised algorithms, we unify the number of clusters set by all algorithms on different datasets to the corresponding number of true cell types. For scGAE, the cluster centers are solved using Spectral and Louvain, and the latent layer clustering results are output. In this study, we use the best Spectral clustering for comparison. There are three classifiers in the scAL, and we choose the default SVM. The scSemiAE uses KNN, k-means, and Louvain clustering methods. In this paper, we compare the results of the k-means clustering algorithm with the best overall performance. Moreover, this algorithm employs two marking ways, labeling the number of cells of each type and labeling the proportion of all samples. We select the former (algorithm default) in the comparison clustering results.

**Clustering results**

Tables 1-3 show the comparison of our model with unsupervised clustering algorithms as measured by ARI, NMI and ACC metrics, respectively. ​Tables 4-6 show the comparison between scSemiAAE and semi-supervised clustering algorithms. Tables 7 displays the clustering results for the large datasets with tens of thousands of sample sizes. Tables 8-10 are model performance with and without the Gaussian mixture model (GMM). Table 11 is the NMI metric for scSemiAAE with different label ratios. Figure 1 demonstrates cluster images for the algorithms based on 6 real datasets. Figure 2 verifies the necessity of Gaussian mixture clustering.

**Table 1.** ARI Metrics for Unsupervised Algorithms

| ARI | scDeepCluster | scDEC | scDSC | scDHA | scGAE | SC3 | scSemiAAE |
| --- | --- | --- | --- | --- | --- | --- | --- |
| 10X_PBMC | 0.6845 | 0.6076 | 0.6279 | 0.4221 | 0.1211 | 0.6133 | **0.7612** |
| human kidney | 0.3375 | 0.3137 | 0.4229 | 0.5567 | 0.1471 | 0.2359 | **0.6045** |
| worm neuron | 0.3724 | 0.3586 | 0.3147 | 0.1478 | 0.1822 | 0.1822 | **0.6536** |
| Human Liver | 0.6964 | 0.5448 | 0.3876 | 0.714 | \ | \ | **0.7473** |
| CITE_PBMC | 0.5141 | 0.5397 | 0.3714 | 0.5952 | \ | \ | **0.6604** |
| Shekhar mouse retina | 0.6254 | 0.4348 | 0.7788 | 0.6894 | \ | \ | **0.8173** |

**Table 2.** NMI Metrics for Unsupervised Algorithms

| NMI | scDeepCluster | scDEC | scDSC | scDHA | scGAE | SC3 | scSemiAAE |
| --- | --- | --- | --- | --- | --- | --- | --- |
| 10X_PBMC | 0.7537 | 0.6971 | 0.6516 | 0.6388 | 0.5203 | 0.7155 | **0.7701** |
| human kidney | 0.5182 | 0.5369 | 0.6464 | **0.6857** | 0.5872 | 0.4228 | 0.6634 |
| worm neuron | 0.506 | 0.6287 | 0.5718 | 0.4695 | 0.5422 | 0.5422 | **0.7311** |
| Human Liver | 0.7897 | 0.6825 | 0.4537 | 0.7787 | \ | \ | **0.8053** |
| CITE_PBMC | 0.7011 | 0.7166 | 0.5233 | 0.7215 | \ | \ | **0.7379** |
| Shekhar mouse retina | **0.7966** | 0.7564 | 0.7132 | 0.6596 | \ | \ | 0.7153 |

**Table 3.** ACC Metrics for Unsupervised Algorithms

| ACC | scDeepCluster | scDEC | scDSC | scDHA | scGAE | SC3 | scSemiAAE |
| --- | --- | --- | --- | --- | --- | --- | --- |
| 10X_PBMC | 0.7497 | 0.6562 | 0.7256 | 0.6144 | 0.1709 | 0.6966 | **0.8452** |
| human-kidney | 0.4724 | 0.4179 | 0.5411 | 0.7171 | 0.21284 | 0.3517 | **0.7576** |
| worm-neuron | 0.4745 | 0.6098 | 0.4895 | 0.4271 | 0.3036 | 0.3036 | **0.7604** |
| Human_Liver | 0.7350 | 0.6289 | 0.5743 | 0.7718 | \ | \ | **0.7848** |
| CITE_PBMC | 0.6910 | 0.6404 | 0.5581 | 0.7140 | \ | \ | **0.7310** |
| Shekhar_mouse_retina | 0.6534 | 0.5847 | 0.7252 | 0.6390 | \ | \ | **0.7511** |

| ARI | scDCC | Itclust | scSemiAE | scAL-SVM | scSemiAAE |
| --- | --- | --- | --- | --- | --- |
| 10X_PBMC | 0.7036 | 0.6477 | 0.6459 | 0.7159 | **0.7612** |
| human kidney | 0.4697 | 0.3612 | 0.4836 | 0.4809 | **0.6045** |
| worm neuron | 0.4273 | 0.5416 | 0.5012 | 0.1315 | **0.6536** |
| Human Liver | 0.6750 | 0.6047 | 0.4622 | 0.7313 | **0.7473** |
| CITE_PBMC | 0.5319 | 0.3934 | 0.4078 | 0.6109 | **0.6604** |
| Shekhar mouse retina | 0.6341 | 0.7324 | 0.6338 | 0.2626 | **0.8173** |

**Table 4.** ARI Metrics for Semi-Supervised Algorithms

**Table 5.** NMI Metrics for Semi-Supervised Algorithms

| NMI | scDCC | Itclust | scSemiAE | scAL-SVM | scSemiAAE |
| --- | --- | --- | --- | --- | --- |
| 10X_PBMC | 0.7498 | 0.6786 | 0.6367 | 0.7463 | **0.7701** |
| human kidney | 0.4697 | 0.3612 | 0.4836 | 0.6480 | **0.6634** |
| worm neuron | 0.6603 | 0.5168 | 0.6569 | 0.3775 | **0.7311** |
| Human Liver | 0.7759 | 0.6528 | 0.6539 | 0.7844 | **0.8053** |
| CITE_PBMC | 0.7218 | 0.5516 | 0.6049 | 0.7183 | **0.7379** |
| Shekhar mouse retina | **0.7901** | 0.6747 | 0.6491 | 0.2321 | 0.7153 |

**Table 6.** ACC Metrics for Semi-Supervised Algorithms

| ACC | scDCC | Itclust | scSemiAE | scAL-SVM | scSemiAAE |
| --- | --- | --- | --- | --- | --- |
| 10X_PBMC | 0.7653 | 0.7550 | 0.7943 | 0.7764 | **0.8452** |
| human kidney | 0.5964 | 0.5603 | 0.6875 | 0.6412 | **0.7576** |
| worm neuron | 0.6243 | 0.6992 | 0.6341 | 0.3356 | **0.7604** |
| HumanLiver | 0.7161 | 0.7038 | 0.5812 | **0.8256** | 0.7848 |
| CITE_PBMC | 0.6334 | 0.5031 | 0.5610 | 0.6716 | **0.7310** |
| Shekhar mouse retina | 0.6723 | 0.6940 | 0.6647 | 0.5009 | **0.7511** |

**Table 7.** Clutering Metrics for Large Datasets

| Datasets | ACC | NMI | ARI |
| --- | --- | --- | --- |
| Shekhar_mouse_retina | 0.7511 | 0.7153 | 0.8173 |
| Tabula Muris | 0.6031 | 0.7456 | 0.5916 |
| Karagiannis | 0.7046 | 0.6859 | 0.5707 |

**Table 8.** NMI Metrics for scSemiAAE

| NMI | scSemiAAE without GMM | scSemiAAE with GMM |
| --- | --- | --- |
| 10X_PBMC | 0.6346 | **0.7701** |
| human kidney | 0.5497 | **0.6634** |
| worm neuron | 0.6159 | **0.7311** |
| Human Liver | 0.7201 | **0.8053** |
| CITE_PBMC | 0.5997 | **0.7379** |
| Shekhar mouse retina | 0.6799 | **0.7153** |
| Baron(human) | 0.6358 | **0.7783** |
| Average | 0.6337 | **0.7431** |

**Table 9.**ACC Metrics for scSemiAAE

| ACC | scSemiAAE without GMM | scSemiAAE with GMM |
| --- | --- | --- |
| 10X_PBMC | 0.6596 | **0.8452** |
| human-kidney | 0.6071 | **0.7576** |
| worm-neuron | 0.6348 | **0.7604** |
| Human_Liver | 0.7102 | **0.7848** |
| CITE_PBMC | 0.6667 | **0.731** |
| Shekhar_mouse_retina | 0.6229 | **0.7511** |
| Baron(human) | 0.6548 | **0.7392** |
| Average | 0.6509 | **0.7670** |

**Table 10.** ARI Metrics for scSemiAAE

| ARI | scSemiAAE without GMM | scSemiAAE with GMM |
| --- | --- | --- |
| 10X_PBMC | 0.6678 | **0.7612** |
| human kidney | 0.5521 | **0.6045** |
| worm neuron | 0.5398 | **0.6536** |
| Human Liver | 0.6152 | **0.7473** |
| CITE_PBMC | 0.5287 | **0.6604** |
| Shekhar mouse retina | 0.7475 | **0.8173** |
| Baron(human) | 0.6617 | **0.7592** |
| Average | 0.6161 | **0.7148** |

**Table 11.** NMI Metrics for scSemiAAE with different label ratios

| NMI | 0% | 5% | 10% | 15% | 20% | 25% |
| --- | --- | --- | --- | --- | --- | --- |
| Baron(human) | 0.6855 | 0.6860 | 0.7256 | 0.7532 | 0.7783 | 0.7814 |
| 10X_PBMC | 0.5864 | 0.6543 | 0.7176 | 0.7489 | 0.7701 | 0.7891 |
| worm-neuron | 0.4153 | 0.5769 | 0.6143 | 0.6312 | 0.6634 | 0.6814 |
| human-kidney | 0.6154 | 0.6578 | 0.6973 | 0.7018 | 0.7311 | 0.7412 |
| Human_Liver | 0.6783 | 0.6899 | 0.7324 | 0.7578 | 0.8053 | 0.8164 |
| CITE_PBMC | 0.6021 | 0.6471 | 0.6752 | 0.715 | 0.7379 | 0.7386 |
| Shekhar_mouse retina | 0.5901 | 0.6189 | 0.7293 | 0.7586 | 0.7153 | 0.7102 |
| Average | 0.5962 | 0.6473 | 0.6988 | 0.7237 | 0.7430 | 0.7511 |

**Figure 1**. Cluster images for scSemiAAE with real scRNA-seq datasets


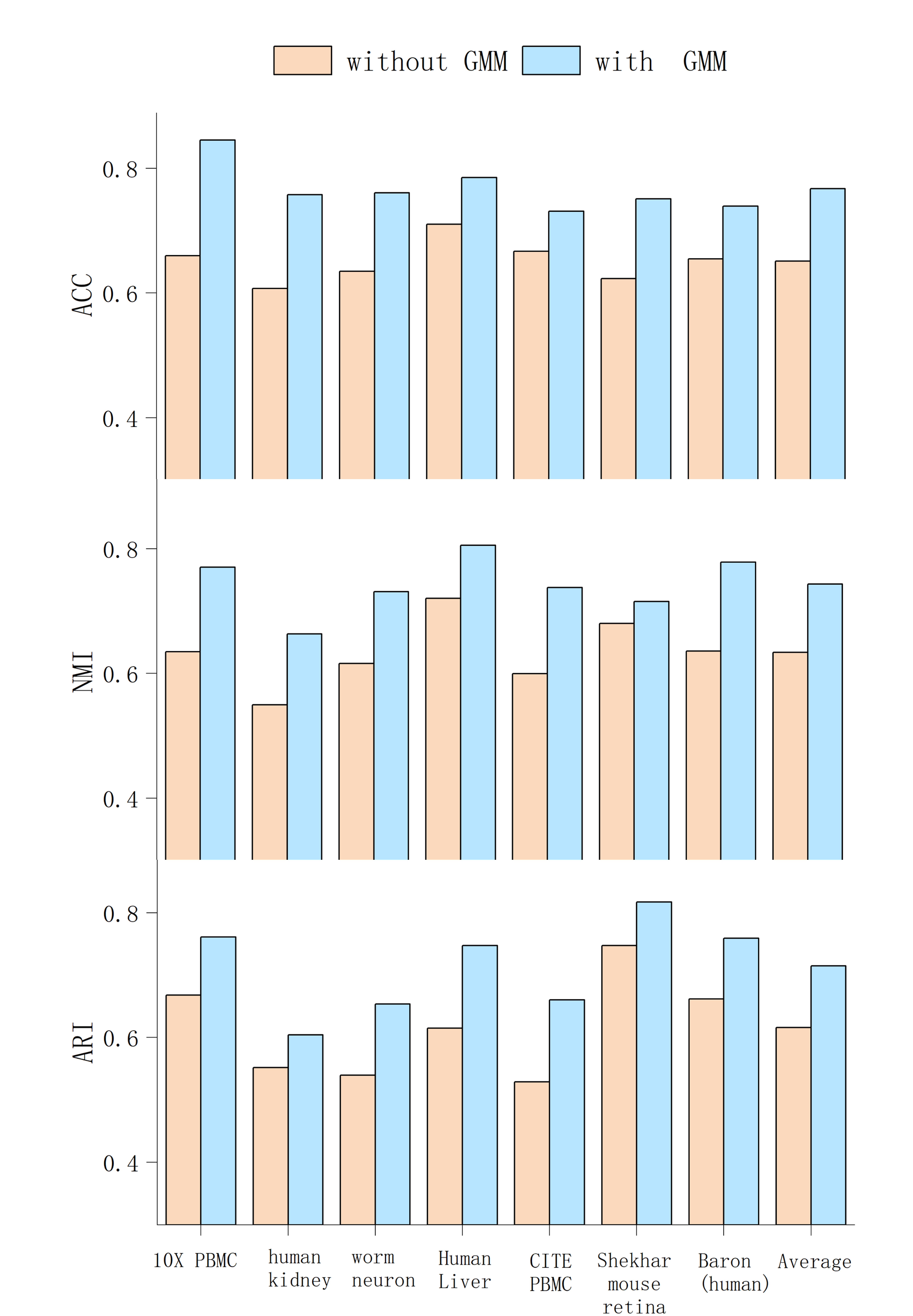


**Figure 2**. Verify the necessity of Gaussian mixture clustering.


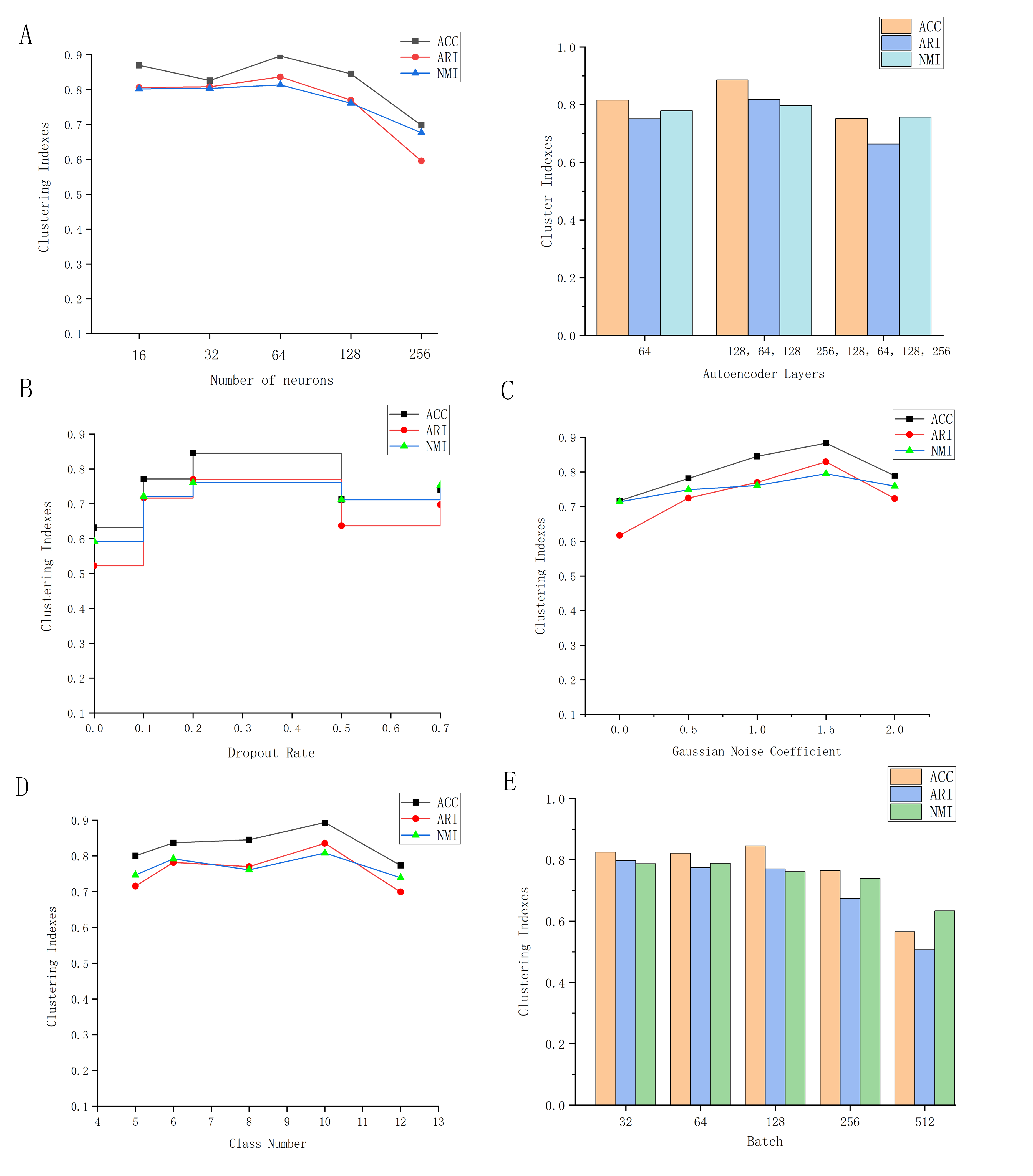
 **Figure 3. Hyperparameter sensitivity analysis.** Parametric analysis of the 10X PBMC dataset based on the clustering metrics ACC, ARI, and NMI. (A) the number of latent layer neurons and the size of the layers in the autoencoder. (B) Dropout rate. (C) Gaussian noise coefficient. (D) Number of clusters. (E) Batch size.

To display the sensitivity variation of the parameters more clearly, we select the 10X PBMC dataset with a small sample size. As shown in Figure 2A, the performance of the clustering index is relatively flat when the number of neurons in the inner layers is small. When the number exceeds 128, the clustering index begins to decline, which may be caused by insufficient dimensionality reduction. For the number of layers, the authors chose 1, 3, and 5 layers to analyze, and it can be seen that the clustering results are better when the number of layers is chosen to be 3. According to Figure 2B and Figure 2C, the size of dropout rate and the Gaussian noise have a certain influence on the noise of the algorithm results, but the clustering indicators are still above 0.6. From Figure 2D and Figure 2E, it is easy to find that scSemiAAE algorithm is robust to parameters such as the number of clusters and batch size.
